# VALIDATION OF GLIM CRITERIA ON MALNUTRITION IN CHINESE OLDER INPATIENTS

**DOI:** 10.1093/geroni/igac059.2715

**Published:** 2022-12-20

**Authors:** Tong Ji, Yun Li, Pan Liu, Yaxin Zhang, Yu Song, Lina Ma

**Affiliations:** Capital Medical University Xuanwu Hospital, Beijing, Beijing, China (People’s Republic); Xuanwu Hospital Capital Medical University, Beijing, Beijing, China (People’s Republic); Xuanwu Hospital Capital Medical University, Beijing, Beijing, China (People’s Republic); Xuanwu Hospital Capital Medical University, Beijing, Beijing, China (People’s Republic); Xuanwu Hospital Capital Medical University, Beijing, Beijing, China (People’s Republic); Xuanwu Hospital Capital Medical University, Beijing, Beijing, China (People’s Republic)

## Abstract

**Backgrounds:**

Malnutrition is a nutritional disorder that has a high incidence and can lead to multiple poor prognoses, such as frailty. Early identification and correct evaluation of malnutrition are essential for improving clinical outcomes. Objective: Therefore, we aimed to explore the applicability and effectiveness of the Global Leadership Initiative on Malnutrition (GLIM) criterion for identifying undernutrition in older inpatients.

**Methods:**

In total, 223 participants aged ≥60 years were involved. Nutrition was evaluateded employing MNA-FF and GLIM criteria, which adopts a two-step procedure. The first step was to use three different methods for nutritional risk screening: NRS-2002, MNA-SF, and MUST. The second step is to diagnose undernutrition. The Clinical Frailty Scale was used to assess frailty. Sensitivity, specificity, Youden index, kappa values, and positive and negative predictive values were measured to test the validity of the GLIM criteria. Logistic regression models were used to assess whether there was a correlation between malnutrition and frailty.

**Results:**

We found that 32.3–49.8% of inpatients were at risk of malnutrition based on the GLIM diagnosis using the three different screening tools, 19.3–27.8% of patients were malnourished. The GLIM criteria with MNA-SF as the diagnostic validation and MNA-FF as a reference showed high consistency (k = 0.629; p < 0.001), sensitivity (90.5%), and specificity (86.4%). Logistic regression analysis showed that malnutrition, using MNA-SF with the GLIM criteria, was relevant to higher likelihood of frailty (OR=1.887; 95% CI: 1.184–2.589). Conclusions: The GLIM criteria with the MNA-SF may be a more reliable malnutrition assessment process in elderly inpatients.

